# Measuring Social Camouflaging in Individuals with High Functioning Autism: A Literature Review

**DOI:** 10.3390/brainsci13030469

**Published:** 2023-03-10

**Authors:** Ivan Mirko Cremone, Barbara Carpita, Benedetta Nardi, Danila Casagrande, Rossella Stagnari, Giulia Amatori, Liliana Dell’Osso

**Affiliations:** Department of Clinical and Experimental Medicine, University of Pisa, 56126 Pisa, Italy

**Keywords:** camouflaging, broad autism phenotype, female autism phenotype, autism spectrum disorder

## Abstract

In the recent years, growing attention has been paid to the use of camouflaging strategies by adult populations suffering from autism spectrum disorder (ASD) with milder manifestations and without intellectual impairment, which may lead to a delay in diagnosis or even a misdiagnosis. In fact, high-functioning ASD individuals were reported to be more aware of their communication difficulties and were more likely make considerable efforts to adjust their behavior to conventional rules of non-autistic individuals, learning to imitate other non-ASD individuals. Moreover, females reported a higher frequency of camouflaging strategies, suggesting a role of camouflaging in the gender gap of the ASD diagnosis. Although camouflaging strategies can sometimes grant a better level of adjustment, even resulting in a hyper-adaptive behavior, they are also often correlated with negative mental health consequences due to the long-term stress associated with continuous attempts to adapt in day-to-day life. In this framework, the aim of the present work was to review the available studies that assessed the presence and correlates of camouflaging strategies in individuals with ASD. Although the literature available on the topic is still scarce, some interesting correlations between camouflaging and anxious and depressive symptoms, as well as suicidality, were highlighted. In particular, the controversial and sometime opposite thoughts and results about camouflaging may be clarified and integrated in light of a dimensional approach to psychopathology.

## 1. Introduction

Autism Spectrum Disorder (ASD) is a heterogeneous neurodevelopmental disorder characterized by a pervasive impairment in reciprocal and social communication and interactions, restricted and repetitive patterns of behavior, interests or activities, lack of socio-emotional reciprocity and impaired sensory integration processing, and can present with different grades of symptom severity and with or without intellectual impairment [1,2]. In the recent years, many studies have focused their attention on evaluating ASD presentation in adulthood, with specific attention on mild manifestations without intellectual impairment that often remain underdiagnosed for many years [3,4], gaining clinical attention after the development of comorbidities and their consequences. The presence of subthreshold autistic traits (AT) was initially reported among first degree relatives of ASD probands, who also seemed to show biochemical and neurobiological alterations similar, although milder, to those of ASD individuals [4,5,6,7,8]. It is now widely recognized how AT are continuously distributed from the clinical to the general population [9,10,11,12,13]. In addition, AT seem to be more frequently detectable in specific populations, including among patients with different kinds of psychiatric disorders, thus leading to the hypothesis that AT may be considered as a vulnerability factor for the development of psychiatric disorders, also increasing the risk for suicidal ideation and behaviors [5,14,15,16,17,18,19,20,21,22]. Noticeably, AT have been associated with a poorer quality of life and with higher suicidality risk, also when subthreshold [10,19,20,23,24,25].

One of the core symptoms of ASD is the impairment in social interactions and communication, and the reduced ability to manage how others may perceive them in social situations [26]. High-functioning ASD individuals (ASD without intellectual disabilities) were reported to be more aware of their communication difficulties, with a low self-perceived social competence which may contribute to the development of depressive and anxious symptoms [27,28]. In particular, to this date, there is strong evidence that people with ASD are more likely to report anxiety symptoms and suffer from anxiety disorders, which can worsen or exacerbate ASD symptoms, create ongoing discomfort, and lead to more behavioral issues [29,30,31,32,33]. Thus, these people will more likely make considerable efforts to adjust their behavior to conventional rules of non-autistic individuals, and subsequently suffer from concurrent psychiatric symptoms due to the long-term stress associated with the continuous attempt to adapt in day-to-day life [27,28,34]. In this framework, the term “social camouflaging” by ASD people is used to indicate a coping strategy for masking difficulties in social situations, and has gained increasing attention in clinical and research settings [35,36,37,38,39,40,41,42,43]. Social camouflaging strategies may include hiding some types of behaviors that might be considered socially unacceptable, or explicitly performing another behavior considered to be more neurotypical in order to appear socially competent [36,43]. Such behaviors may be learned by imitating other peers or even by programmatically studying different kinds of media, and may range from intentionally maintaining eye contact, to adjusting facial expressions and gestures and even using prepared sentences or entire scripts during conversations [34]. Social camouflaging may be partially unintentional, gaining more and more layers of complexity over the years, especially when continuously used since early age. In some cases, it may also lead to the construction of a new identity, sometimes referred to as a “mask”, which the individual adopts in social environments [43].

In the recent literature, the role of social camouflaging in the gender gap related to ASD has been widely discussed. In particular, the systematic use of camouflaging has been considered to be one of the factors responsible for the delay or the misdiagnosis of ASD among females [44,45,46,47,48], hence contributing to the commonly observed male preponderance in ASD prevalence [34]. In this framework, the recent literature stressed how the strikingly higher prevalence of ASD among males should be reconsidered in light of gender specific manifestations of ASD, while, to date, ASD diagnostic criteria are based on the classical male manifestations of the disorder [34,41,49,50]. Besides showing different kinds of restricted interests, including a frequent focus on food and diet [51], females on the autism spectrum often show lower social difficulties, also due to a higher tendency towards the use of camouflaging strategies [52,53]. It should be noted that while epidemiological studies on ASD typically report a male/female ratio around 4:1 [54], the gender gap is reduced to 2:1 in individuals with an intelligence quotient (IQ) lower than 70 points and even more among individuals with greater intellectual disability [55]. On the other hand, among cases without intellectual impairment, the sex ratio may be higher than 5.5:1 [54]. Considering that social camouflaging is typically adopted by high-functioning individuals, the parallel decrease between IQ and gender gap in ASD diagnosis may eventually support the hypothesis of an involvement of the use of camouflaging strategies in the under-diagnosis of ASD among females. Moreover, females are often diagnosed with ASD later in life compared to males [56,57,58,59,60], unless they show comorbid cognitive or behavioral symptoms [45].

Globally, the interest in understanding and detecting camouflaging strategies among individuals with ASD lies in the fact that while they may actually allow a better adjustment to the social environment, including high-standing results in specific fields, on the other hand, they may mask the ASD diagnosis, preventing the individual from receiving proper clinical attention and help. In addition, as reported above, social camouflaging is associated with the presence of greater stress and increased anxiety and depression, also due to the continuous efforts to adjust, which may lead to exhaustion and also interfere with the development of a self-identity [36,43,61,62,63,64,65].

To this date, the literature in this area is still fairly young, and more has to be understood about the specifics of these tactics, the reasons for their adoption, and how they relate to psychiatric symptoms, also due to the lack of a proper conceptual model for camouflaging and the presence of only one validated questionnaire. In this framework, the aim of the present work was to review the available studies that assessed the presence and correlates of camouflaging strategies in individuals with ASD, and raised awareness of a modern spectrum approach to psychopathology that reflects the actual camouflaging experiences of people with ASD—and also takes into account the specific female presentations that often remain undetected—ultimately clarifying the controversial, and sometimes opposite, thoughts towards camouflaging.

## 2. Methods

A literature search was conducted from 1 July to 3 September 2022, using the electronic databases PubMed and Scopus. The following search terms, without filters, restriction or limits were used to identify all potentially eligible records: “camouflaging” “camouflaging AND autism spectrum disorder” “camouflaging AND autism” “camouflaging AND autistic traits” “camouflaging AND high functioning autism”.

The criteria used to include studies in this review were as follows:

1. Human studies;
2. Studies that included only individuals of ages > 18;
3. Articles available in English.

## 3. Attempting to Measure Camouflaging: State of the Art

During the last years, the literature highlighted the need for the development of instruments designed to investigate camouflaging behaviors in clinical and sub-clinical populations, with various proposed methods [42,65,66,67].

The first studies in the field were led through ad hoc questions, such as questions regarding a person’s feeling of “acceptance” by society (“how accepted by society do you feel as an autistic person?”) [68] or through indirect evaluation, such as by assessing the difference between self-perceived autistic traits and the external perception of them [41]. Besides anecdotal descriptions and case reports [42], one of the first studies in the field was led by Bargiela et al. [64], who reported the qualitative presence of the feeling of exhaustion and a negative impact on the self-identity linked to the use of camouflaging strategies in 14 late-diagnosed ASD women narrating their stories. All participants were evaluated with a semi-structured interview that began with asking participants to tell the story of how they were diagnosed with ASD and then moved to consideration of interests, social relationships, sensory experiences and mental health. The Autism Quotient (AQ) was used to confirm the clinical diagnosis of ASD, the General Health Questionnaire-12 (GHQ-12) to assess the current mental state, the Hospital Anxiety and Depression Scale (HADS) to detect depression and anxiety symptoms and the Wechsler Test for Adult Reading (WTAR) for evaluating intelligence. In the same year, Tierney et al. [69] interviewed ten female adolescents with a diagnosis of ASD using interpretative phenomenological analysis (IPA) to explore their experiences in social relationships. The results showed the difficulty in maintaining friendships during adolescence, leading them to develop strategies such as masking and imitation. Dean et al. [70] stressed how the male framework may make it easier to understand the social challenges of boys with ASD, emphasizing a male bias in our perception of ASD (See Table 1).

Lai et al. [41] tried to indirectly explore the correlation between camouflaging behaviors, and anxious and depressive symptoms in a sample composed of 30 females and 30 males with an ASD diagnosis. Participants were assessed using the Autism Diagnostic Observation Schedule (ADOS), the AQ score and Reading the Mind in the Eyes test (RMET). Measures of camouflaging were obtained through the difference between external behaviors, and both self-rated autistic-like traits and mentalizing ability. Anxiety and depression were assessed using Beck Anxiety Inventory (BAI) and Beck’s Depression Inventory (BDI). The results showed a gender difference: ASD women scored significantly higher than men on camouflage. Moreover, camouflaging scores were positively correlated with BDI but not BAI total score, and the association between camouflaging and BDI score was significant exclusively in the male population, expressing the presence of more depressive symptoms.

Cage et al. [68] led instead a qualitative study assessing 111 ASD adults with the Depression, Anxiety and Stress Scale (DASS-21) and ad hoc autism acceptance questions. For example, participants were asked if the society generally accepted them; they were asked to rate the statement “over the past week, I have felt accepted by society as an autistic person/person with autism” on a 5-point scale; they were also asked “how much have you personally accepted yourself as an autistic person”. Their results highlighted that participants who spontaneously reported camouflaging-like behaviors showed greater symptoms of depression.

Another qualitative study came from Hull et al. [42], who evaluated camouflaging experiences in 92 ASD adults through a newly designed questionnaire for camouflaging, investigating the motivation and consequences of the use of these strategies. The authors tried to identify key elements of camouflaging, which, according to their results, seemed to feature both masking and compensation behaviors. Moreover, the main motivations for camouflaging featured attempts to increase connections with others and to fit in, while most of the participants reported that the main consequences of camouflaging were mental, physical and emotional exhaustion, feelings of extreme anxiety and stress, together with an impaired self-perception and identity.

Noticeably, camouflaging was associated also with suicide risk. Cassidy et al. [71] assessed a sample of 164 ASD adults with four questions designed to quantify tendency to adopt camouflaging strategies. Results showed that suicidality, measured through the Suicide Behaviors Questionnaire-Revised (SBQ-R), was significantly correlated with camouflaging; interestingly, the latter appeared to be an even greater predictor of suicidality than the delay in ASD diagnosis. The study also reported no differences in camouflaging behaviors between ASD males and females (See Table 2).

In this framework of increasing interest towards social camouflaging, during 2019, Hull et al. [72] developed and validated the “Camouflaging Autistic Traits Questionnaire” (CAT–Q), which, to the best of our knowledge, still remains the only available instrument for measuring this condition. An Italian version is also available [73]. The questionnaire is composed of 25 items, whose answers are organized in a seven-point Likert scale and are divided into three domains: *Compensation*, which includes nine items and aims to investigate actively compensative behaviors put in place to adjust to social situations; *Masking*, which includes eight items and measures the habit of hiding autistic-like features; and *Assimilation*, which contains eight items and investigates the strategies used in attempting to fit in with others during social interactions. Both the English and the Italian version showed excellent internal consistency, test re-test reliability and convergent validity with other instruments assessing the autism spectrum [72,73]. The validation study of the CAT-Q was conducted in 354 ASD participants and 478 non-ASD adults. The instrument reported very good internal consistency and reliability. The exploratory factor analysis identified a three-factor structure, consisting of Compensation, Masking and Assimilation [72]. The same questionnaire was used in another study by Hull et al. [74] for evaluating gender differences in camouflaging behaviors among autistic and non-autistic adults. The results highlighted that autistic females (*n* = 182) scored higher than males (*n* = 108) on two of three CAT-Q subscales: *Masking* and *Assimilation*, but not on the *Compensation* subscale. However, no gender difference was reported among non-autistic participants (252 females vs. 193 males).

In another study by Cassidy et al. [75], the authors focused again on the link between camouflaging and suicidality, investigating a non-clinical sample of 160 undergraduate students with the Autism Spectrum Quotient-Short (AQ-S), the CAT-Q, the Interpersonal Needs Questionnaire (INQ-15), the Patient Health Questionnaire-9 (PHQ-9), the General Anxiety Disorder-7 (GAD-7) and the SBQ-R. The results showed that camouflaging behaviors were associated with an increased risk of lifetime suicidality and of experiencing thwarted belongingness (see Table 3).

Cage et al. [76] focused instead more generally on the consequences of protracting camouflaging strategies in a sample of 262 ASD participants using the CAT-Q and the DASS 21. The sample was later divided into three subgroups: high camouflagers (who reported higher camouflaging scores in both formal and interpersonal contexts), switchers (who showed higher levels of camouflaging in one context) and low camouflagers (low score in both contexts). The results showed how switchers and high camouflagers showed similar levels of anxiety and stress symptoms, which were significantly higher compared to the low camouflagers. Interestingly, even though depressive symptom severity was higher compared to the mean score of the non-ASD population, no differences in them were found between the three groups. In addition, they found that female participants used camouflaging strategies for conventional reasons more often than male participants did, indicating a significant difference between the sexes for conventional reasons but not for relational ones. Another study focused on analyzing camouflaging outcomes in a non-clinical sample [77]. The authors evaluated a group of 58 women with a high score on the Broad Autism Phenotype Questionnaire (BAPQ) and whose autistic traits may have potentially remained underdiagnosed also due to the use of camouflaging strategies. The results showed that the majority of the sample reported some form of psychological distress (depression/anxiety), evaluated with DASS-21, suicidality, evaluated with SBQ-R, and reduced daily functioning, evaluated with WHODAS 2.0, although only CAT-Q total scores, and not generally AT, were significantly correlated with psychological distress. In addition, in a subsample with high CAT-Q scores, camouflaging was also significantly correlated with suicidality and reduced daily functioning. On the basis of these data, the authors suggested that the association between camouflaging and mental health may be linked to the effort of camouflaging and not to the severity of the AT being camouflaged [72] (see Table 4).

Robinson et al., in 2020 [78], studied a sample of 278 autistic and 230 typically developing participants. The study explored the relationship between the five-factor model of personality (using The Big Five Inventory), trait Emotional Intelligence (EI, using Trait Emotional Intelligence Questionnaire), and camouflaging (using CAT-Q) in autistic and non-autistic individuals. In the typical developing sample, there were negative correlations between camouflaging and agreeableness, extraversion, and trait EI. A positive correlation was found between neuroticism and camouflaging, and a negative correlation between conscientiousness and camouflaging. However, only the relationship between neuroticism and camouflaging was significant in the autistic sample. Despite no overall association between CAT-Q total score and extraversion in the autistic sample, a significant correlation was found with the camouflaging subscale of assimilation. In a study by Perry et al. [79], questions concerning perceived autism-related stigma, individualistic and collective strategies, camouflaging and mental wellbeing were posed to 223 ASD adults. They used The Stigma Consciousness Scale, Nario-Redmond et al.’s 13-item measure of individualistic strategy use adapted, Nario-Redmond et al.’s 13-item measure of collective strategy use adapted, the CAT-Q and The Warwick–Edinburgh Mental Wellbeing Scale. The stigma associated with ASD and the employment of both individualistic and group strategies were positively correlated with higher camouflaging. Lower wellbeing was linked to autism stigma, although camouflaging did not mitigate this association. In a later study, Hull et al. [80] proceeded to explore the association between camouflaging, anxiety and depression among 305 adults with ASD. Participants were assessed with CAT-Q, BAPQ, Social Anxiety Scale (LSAS), the GAD 7 and PHQ-9. The results highlighted that greater camouflaging scores predicted greater scores in measures of generalized anxiety, depression, and social anxiety in a significant manner, independently of age and levels of AT. Another study from Hull et al. [81] focused on the possible indicators of self-reported camouflaging in ASD adolescents. The sample was composed of 58 ASD adolescents (F = 29, M = 29; age: 13–18 years) assessed with the CAT-Q; the Behavior Rating Inventory of Executive Function, Second Edition (BRIEF-2), as a measure of executive function difficulties; the “Strange stories”, a semi-naturalistic measure of the Theory of Mind (ToM); the WASI-II, a standardized measure of intellectual ability; and the Social Reciprocity Scale (SRS), a standardized parent-report measure of a child’s autistic symptoms. None of the self-reported camouflaging scores were correlated with age, intelligence or ToM. Although the observed effect was small, executive function issues were inversely correlated with self-reported total and masking scores on the CAT-Q [81]. In the validation study of the Italian version of the CAT-Q, the authors also confirmed the association between camouflaging behaviors and autistic traits as measured by the Adults Autism Subthreshold Spectrum (AdAS Spectrum) questionnaire in a sample of university students and workers [73]. A further investigation in Italy [82] explored the prevalence of autistic traits and camouflaging behaviors in a population of university students. Autistic traits were assessed by means of the AdAS Spectrum questionnaire. A total of 2141 students were enrolled in the study. The sample was divided between participants with different levels of autistic traits depending on the scores on the AdAS Spectrum and, on the basis of the followed academic courses, into four broad categories—pure sciences; applied sciences; humanities and economics; law and political sciences—in order to compare students from various academic backgrounds. The results revealed a significant main influence of both academic field and sex on AdAS Spectrum domain scores. When compared to the “Applied Sciences” group, “Pure sciences” students demonstrated significantly higher AdAS Spectrum and CAT-Q scores.

The same study also highlighted that when sex differences were taken into account, males considerably outperformed females on the AdAS overall scores. Participants with higher autistic traits showed higher CAT-Q scores, but no gender differences were reported for camouflaging strategies. The authors hypothesized that the lack of gender difference in camouflaging behaviors reported in their sample may be linked to the specific population investigated. Due to the highly demanding university environment, it would be possible that individuals with autistic traits, but lower camouflaging abilities, may be less represented in academic populations [82]. McQuaid et al. [83] assessed instead a sample of ASD adults (*n* = 502; age 18 to 49), using the CAT-Q, aiming to evaluate the relationship between camouflaging and sex, gender identity (gender diverse vs. cisgender) and diagnostic timing (childhood/adolescent-diagnosed vs. adult-diagnosed). Compared to ASD males, ASD females reported more camouflaging on the CAT-Q. Compared to cisgender people, gender diverse adults reported more camouflaging on the Compensation subscale. Individuals who were diagnosed as adults showed higher Assimilation and Compensation than those who were diagnosed as children or adolescents. Finally, a recent work [84] also highlighted that ASD females reported a higher frequency of camouflaging strategies. The sample utilized was composed of young people with an ASD diagnosis (*n* = 78), with high autistic features but no diagnosis (*n* = 177), or low autistic traits (*n* = 180), assessed with the CAT-Q, the Social Responsiveness Scale-Second Edition (SRS-2) for autistic traits, the Friendship Quality Scale (FQS) as a measure of friendship relationships, the UCLA loneliness scale to evaluate loneliness as a potential cause, indicator, or effect of camouflaging, the subjective happiness scale, the World Health Organization Quality of Life Scale (WHO-QoL Bref) and the Strengths and Difficulties Questionnaire (SDQ) for emotional and behavioral difficulties. The results reported that in the diagnosed group, camouflaging significantly predicted a lower psychological quality of life, while in the high traits group, it predicted a lower social quality of life. Moreover, the CAT-Q total score significantly predicted lower social quality of life for high trait males, but not for high trait females (see Table 5).

## 4. Discussion

The research presented highlights the association between camouflaging and suicidality, which has also been stressed in both clinical and non-clinical populations, in line with the literature which has stressed the presence of an increased suicidal risk also among individuals with sub-clinical autistic traits [17,74]. However, the literature in this field is still in its infancy and the specific nature of these strategies and the motivations at the basis of their use, as well as their correlations with psychiatric symptoms, need to be better clarified [39]. While it may be hypothesized that camouflaging behaviors could be considered as a coping strategy in response to feelings of social isolation, perceived social difficulties, low self-esteem and acceptance; on the other hand, in a vicious cycle, they may also contribute to the worsening of the same symptoms they are possibly meant to prevent, with negative consequences such as feelings of exhaustion, distress and a disruptive impact on self-image and identity [44,76]. In conclusion, the specific causal or temporal relationship between camouflaging and stress, anxiety, or depression remains controversial and should be further investigated by longitudinal studies.

In the last decade, growing attention has been paid to the use of camouflaging strategies in autism populations, and to its correlation with quality of life and psychiatric outcomes. Although camouflaging strategies might grant a better level of adjustment, resulting, in some cases, in hyper-adaptation in particular fields, they are also often correlated with negative mental health consequences. As the studies reviewed herein seem to suggest, camouflaging strategies, although adopted by individuals with the aim to improve social inclusion, are also significantly correlated with psychological distress. In particular, a correlation emerged between those who have a higher tendency to camouflage and the presence of symptoms of depression or anxiety [74,76,77], even after accounting for the severity of autistic traits.

Another issue that needs to be clarified in the field of social camouflaging is that of gender difference. The first reports of camouflaging were reported among women, and the increasing focus on this behavior in the scientific literature was also due to its hypothesized link with the under-recognition of autism in females [38,59]. Moreover, although controversial, when considering the growing number of studies that stressed the presence of possible different female presentations of ASD, which may be misdiagnosed with other conditions including anorexia nervosa or borderline personality disorder, we could hypothesize that camouflaging strategies may also have a role in favoring and perseverating such misdiagnoses in female ASD individuals [24,85,86,87].

However, the suggested higher prevalence of camouflaging strategies among females was not always confirmed, seeming to vary depending on the specific sample and environment [72,75,82]. The specific link between camouflaging and anxiety/depressive symptoms is also a feature that, according to some authors, may show gender differences, thus needing to be better investigated [44]. Many questions still need to be answered; however, progress in this field is limited by the lack of a proper conceptual model for camouflaging and the presence of only one validated questionnaire [72,73]. Reaching a shared consensus on the theoretical conceptualization of social camouflaging—as a feature specific to, but possibly not exclusive of, people on the autism spectrum—should be considered as one of the next goals to aim for. Such a model should reflect the actual camouflaging experiences of people with ASD, taking into account the specific female presentations that often remain undetected due to the actual gender biased conceptualization of ASD [43,46,64,72,82,88,89,90].

Moreover, the model should be developed in light of a spectrum approach to psychopathology; while autism spectrum symptoms and traits could be considered continuously distributed from the general to the clinical populations, camouflaging strategies may as well be conceptualized as a continuum, from light tendencies to a full-blown maladaptive coping strategy [3,4,43,80]. A spectrum approach may also help in clarifying the controversial, and sometime opposite, thoughts towards camouflaging. Meanwhile, as reported above, many authors stressed that the prolonged use of these strategies may have mental health implications; other clinicians, on the contrary, may suggest the theoretical usefulness of camouflaging through promoting interventions for treating ASD social difficulties [20]. While the presence of a non-pathological, possibly adaptive dimension of camouflaging could be hypothesized, the impact of these efforts on the individual’s well-being should be carefully considered, and the active promotion of camouflaging should be regarded with caution [20,36,59,67]. Starting with a shared comprehensive model, future research will be able to answer the many questions still pending regarding social camouflaging, such as the individual, gender and age-related differences, its variability during the lifetime, and the long-term outcomes related to global functioning, achievements and quality of life [42].

## 5. Limits

This review should be considered in light of some limitations. First of all, this is a narrative review, so it lacks the systematicity and reproducibility of a systematic one. Secondly, to this date, there is only one validated questionnaire for the investigation of camouflaging strategies. Thirdly, the influence of gender, although frequently cited, seems to vary depending on the specific sample and environmental factor. Lastly, the literature available is still limited and results reported are controversial, and sometimes even opposite.

Moreover, we recognize the presence of two previous reviews assessing camouflaging in the frame of ASD [40,91]. While the first is a systematic review on the whole autism spectrum and camouflaging strategies [40], the second aims to compare different methods for measuring camouflaging in autism [91]. Unlike these previous works, our article aims to give a detailed narrative description of the actual literature available on camouflaging in individuals with high functioning autism and proposes a modern spectrum approach to the topic that could help to achieve a consensus on many aspects of this controversial topic.

## 6. Conclusions

Despite the limited literature available, the lack of a proper conceptual model for camouflaging, the presence of only one validated questionnaire, and the controversial causal or temporal relationship, some interesting correlations between camouflaging, anxious and depressive symptoms and suicidality were highlighted.

However, many aspects of this dimension remain controversial and should be further investigated by longitudinal studies and integrated in light of a dimensional approach to psychopathology. In particular, we deem worthy of further study the correlation between camouflaging and quality of life and psychiatric outcomes, either positive or negative, with deeper insight into gender differences.

In conclusion, one of the next objectives to aim for should be achieving consensus on the theoretical conception of social camouflaging as a trait unique, but potentially not exclusive, to those on the autistic spectrum.

## Figures and Tables

**Table 1:** The available literature on Social Camouflaging (Part 1).

Ref.	Sample	Methods	Results
Bargiela et al. (2016) [61]	ASD (F = 14, from 18 to 35 year)	AQ-10; GHQ-12; HADS; WTAR	Feeling of exhaustion and a negative impact on self-identity after camouflaging.
Tierney et al. (2016) [69]	ASD (F = 10, mean age 14.4 ± 1.04)	Semi-structured interviews; IPA	Psychological health suffers from the use of camouflage tactics and since their problems were concealed, it is possible that it makes it more difficult for them to get help.
Dean et al. (2016) [70]	ASD (F = 24 mean age 7.75 ± 1.22; M = 24 mean age 7.71 ± 1.23); TD (F = 24; M = 24)	ADOS; SB-5; POPE	Girls with ASD have different social experiences than boys with ASD.

AQ-10: Autism Spectrum Quotient (10 items); GHQ-12: General Health Questionnaire-12; HADS: Hospital Anxiety and Depression Scale; WTAR: Wechsler Test for Adult Reading; IPA: Interpretative Phenomenological Analysis; ADOS: Autism Diagnostic Observation Schedule; SB-5: The Stanford–Binet Intelligence Scale: Fifth Edition; POPE: The Playground Observation of Peer Engagement.

**Table 2:** The available literature on Social Camouflaging (Part 2).

Ref.	Sample	Methods	Results
Lai et al. (2016) [41]	ASD (F = 30 mean age: 27.2 ± 7.3; M = 30 mean age 27.8 ± 7.6)	ADOS; AQ; RMET; BAI; BDI	Higher score of CF in ASD females; CF positively correlated with total score of BDI exclusively in male ASD.
Cage et al. (2017) [68]	ASD = 111 (M = 27; F = 79; other = 14; mean age 36.4 ± 12.0)	DASS-21	Greater symptoms of depression in participants who spontaneously report camouflaging.
Hull et al. (2017) [42]	ASD (F = 55 mean age 36.98 ± 14.21; M = 30 mean age 41.03 ± 18.08; other = 7 mean age 32.67 ± 9.25)	43 questions on motivations, characteristics, consequences and attitudes towards camouflaging	Mental, physical and emotional exhaustion were the main consequence of camouflaging alongside feelings of extreme anxiety and stress.
Cassidy et al. (2018) [71]	ASD = 164 (M = 65 mean age 41.52 ± 11.73; F = 99 mean age 38.89 ± 10.47); HC 169 (M = 54 mean age 39.11 ± 10.09; F = 115 mean age 41.48 ± 11.18)	Four questions to quantify tendency to adopt CF strategies; AQ; NSSI; SBQ-R	Significant correlation between CF and suicidality. No differences in CF behavior between male and female ASD.

ADOS: Autism Diagnostic Observation Schedule; AQ Autism Spectrum Quotient; RMET: Reading the Mind in the Eyes test; BAI: Beck Anxiety Inventory; BDI: Beck’s Depression Inventory; DASS-21: depression anxiety stress scale-21; NSSI: Non-suicidal self-injury; SBQ-R: Suicide Behaviors Questionnaire-Revised.

**Table 3:** The available literature on Social Camouflaging (Part 3).

Ref.	Sample	Methods	Results
Hull et al. (2019) [72]	ASD = 354 (M = 108; F = 179; other =17; not stated = 50; mean age 41.93 ± 13.55); Non-ASD = 478 (M = 192; F = 255; other = 29; not stated = 2; mean age 30.24 ± 13.72; Expl. sample = 402 (M = 139; F = 246; gender = 17; not stated = 0; mean age 37.02 ± 15.02); Conf. sample = 430 (M = 161; F = 188; gender = 29; not stated = 52; mean age 35.15 ± 14.21)	CAT-Q; BAPQ; LSAS; WEMWBS; PHQ-9; GAD-7	Positive correlations between the CAT-Q and measures of social anxiety, anxiety, and depression; negative correlation between the CAT-Q and wellbeing.
Hull et al. (2020) [74]	ASD = 306 (M = 108 mean age 46.68 ± 13.98; F = 182 mean age 39.91 ± 12.75; non-binary = 16 mean age 33.50 ± 11.74) Non-ASD = 472 (M = 193 mean age 30.94 ± 14.78; F = 252 mean age 29.86 ± 13.40; non-binary = 27 mean age 26.52 ± 10.74)	CAT-Q; BAPQ	Autistic females score higher than males on two of three CAT-Q subscales. No gender difference among non-autistic participants.
Cassidy et al. (2020) [75]	160 (M = 21, F = 139, from 18 to 23 years)	AQ-S; CAT-Q; the INQ-15; PHQ-9; GAD-7; SBQ-R	CF total scores predictors of lifetime suicidality.

CAT-Q: Camouflaging Questionnaire; BAPQ: Broad Autism Phenotype Questionnaire; LSAS: Social Anxiety Scale; WEMWBS: Warwick–Edinburgh Mental Wellbeing Scale; PHQ-9: Patient Health Questionnaire; GAD-7: Generalized Anxiety Disorder; AQ-S: Autism Spectrum Quotient-Short; INQ-15: Interpersonal Needs Questionnaire; SBQ-R: Suicide Behaviors Questionnaire-Revised.

**Table 4:** The available literature on Social Camouflaging (Part 4).

Ref.	Sample	Methods	Results
Cage et al. (2019) [76]	ASD 262 (M = 111; F = 135; Other = 16; mean age 33.62 ± 11.52)	CAT-Q; DASS-21; RAADS-14	Higher anxiety and stress symptoms in switchers and high camouflagers. Higher depression levels in all groups, compared to the mean score in the non-ASD population.
Beck et al. (2020) [77]	High BAPQ (F = 58 mean age 27.15 ± 6.17)	BAPQ; DASS-21; SBQ-R; WHODAS 2.0; ADOS-2; SRS-2; AQ; CAT-Q; WASI-II	Psychological distress, suicidality and reduced daily functioning in all samples. Significant correlation between CAT-Q total scores and distress. In a subsample, high CAT-Q scores significantly correlate with suicidality and reduced daily functioning.

CAT-Q: Camouflaging Questionnaire; DASS-21: depression anxiety stress scale-21; RAADS-14: Ritvo Autism and Asperger Diagnostic Scale; BAPQ: Broad Autism Phenotype Questionnaire; SBQ-R: Suicide Behaviors Questionnaire-Revised; WHODAS 2.0: World Health Organization Disability Assessment Schedule, Second Edition; ADOS-2: Autism Diagnostic Observation Schedule, Second Edition, Module 4; SRS-2: Social Responsiveness Scale, Second Edition, Adult Self-Report; AQ: Autism Spectrum Quotient; WASI-II: Wechsler Abbreviated Scale of Intelligence, Second Edition.

**Table 5:** The available literature on Social Camouflaging (Part 5).

Ref.	Sample	Methods	Results
Robinson et al. (2020) [78]	592 (ASD 268) (F = 404; M = 172; mean age = 36.8 years ± 15.4)	BAPQ; BFI; TEIQue-SF; CAT-Q	Trait EI negatively associated with autistic traits in both autistic and TD samples. Differences between autistic and TD groups in the predictors of camouflaging.
Perry et al. (2022) [79]	ASD 223 (F = 130; M = 53; non-binary or other gender terminology = 39; not to say = 1; mean age 34.19 ± 11.00)	Individualistic Strategy Use; Collective Strategy Use; Stigma Consciousness Scale; CAT-Q; WEM-WBS; RAADS-14	Higher levels of self-reported camouflaging predicted by higher levels of individualistic and collective strategy use, as well as higher levels of perceived stigma against autism. Autism-related stigma has a negative relationship with mental wellbeing.
Hull et al. (2021) [80]	ASD 305 (M = 104; F = 181; other = 18, from 18 to 75 years)	CAT-Q; BAPQ; LSAS; GAD-7; PHQ-9	CF score significantly predicts greater scores in measures of generalised anxiety, depression, and social anxiety.
Hull et al. (2021) [81]	ASD 58 (F = 29; M = 29, 13–18 years, mean age 14.48 ± 1.74)	CAT-Q; BRIEF-2; Strange stories; WASI-II; SRS	Fewer executive function difficulties predict greater use of total camouflaging strategies and the compensation subscale, but not the masking or assimilation subscales.
Dell’Osso et al. (2022) [82]	2141 (F = 1415; M = 726)	AdAS spectrum; CAT-Q	Higher presence of camouflaging among individuals in the more severe range of the autism spectrum.
McQuaid et al. (2022) [83]	ASD (502, from 18 to 49 years)	AQ-28; CAT-Q	ASD females report more camouflaging across CAT-Q subscales compared to males.
Milner et al. (2022) [84]	ASD (78), high autistic features but no diagnosis	CAT-Q; SRS-2; FQS; UCLA loneliness scale;	In individuals with ASD, camouflaging significantly predicts a lower psychological quality of life; in the high traits group, it predicts a lower social quality of life. The CAT-Q total score predicts lower social quality of life for high trait males, but not for high trait females.

BAPQ: Broader Autism Phenotype Questionnaire; BFI: Big Five Inventory; TEIQue-SF: Trait Emotional Intelligence Questionnaire–Short Form; CAT-Q: Camouflaging Questionnaire; WEM-WBS: Warwick–Edinburgh Mental Wellbeing Scale; RAADS-14: Ritvo Autism and Asperger Diagnostic Scale; LSAS: Social Anxiety Scale; GAD-7: Generalized Anxiety Disorder; PHQ-9: Patient Health Questionnaire; BRIEF-2: Behavior Rating Inventory of Executive Function, Second Edition; WASI-II: Wechsler Abbreviated Scale of Intelligence, Second Edition; SRS: Social Responsiveness Scale; AdAS: Adult Autism Spectrum questionnaire; AQ-28: the 28-item Autism-Spectrum Quotient; SRS-2: Social Responsiveness Scale, Second Edition; FQS: Friendship Quality Scale.

## Data Availability

All data generated or analyzed during this study are included in this published article.

## References

[1] American Psychiatric Association (2013). Diagnostic and Statistical Manual of Mental Disorders: DSM-5.

[2] American Psychiatric Association (2022). Diagnostic and Statistical Manual of Mental Disorders: DSM-5-TR.

[3] Dell’Osso L., Dalle Luche R., Gesi C., Moroni I., Carmassi C., Maj M. (2016). From Asperger’s autistischen psychopathen to DSM-5 autism spectrum disorder and beyond: A subthreshold autism spectrum model. Clin. Pract. Epidemiol. Ment. Health.

[4] Dell’Osso L., Gesi C., Massimetti E., Cremone I.M., Barbuti M., Maccariello G., Moroni I., Barlati S., Castellini G., Luciano M. (2017). Adult Autism Subthreshold Spectrum (AdAS Spectrum): Validation of a questionnaire investigating subthreshold autism spectrum. Compr. Psychiatry.

[5] Sucksmith E., Allison C., Baron-Cohen S., Chakrabarti B., Hoekstra R.A. (2013). Empathy and emotion recognition in people with autism, first-degree relatives, and controls. Neuropsychologia.

[6] Carpita B., Muti D., Dell’Osso L. (2018). Oxidative Stress, Maternal Diabetes, and Autism Spectrum Disorders. Oxidative Med. Cell. Longev..

[7] Carpita B., Marazziti D., Palego L., Giannaccini G., Betti L., Dell’Osso L. (2020). Microbiota, Immune System and Autism Spectrum Disorders: An Integrative Model towards Novel Treatment Options. Curr. Med. Chem..

[8] Afif I.Y., Manik A.R., Munthe K., Maula M.I., Ammarullah M.I., Jamari J., Winarni T.I. (2022). Physiological Effect of Deep Pressure in Reducing Anxiety of Children with ASD during Traveling: A Public Transportation Setting. Bioengineering.

[9] Baron-Cohen S., Wheelwright S., Skinner R., Martin J., Clubley E. (2001). The autism-spectrum quotient (AQ): Evidence from Asperger syndrome/high-functioning autism, males and females, scientists and mathematicians. J. Autism Dev. Disord..

[10] Skylark W.J., Baron-Cohen S. (2017). Initial evidence that non-clinical autistic traits are associated with lower income. Mol. Autism.

[11] Suzuki T., Miyaki K., Eguchi H., Tsutsumi A. (2018). Distribution of autistic traits and their association with sociodemographic characteristics in Japanese workers. Autism.

[12] Dell’Osso L., Carpita B., Cremone I.M., Muti D., Diadema E., Barberi F.M., Massimetti G., Brondino N., Petrosino B., Politi P. (2019). The mediating effect of trauma and stressor related symptoms and ruminations on the relationship between autistic traits and mood spectrum. Psychiatry Res..

[13] Carpita B., Muti D., Muscarella A., Dell’Oste V., Diadema E., Massimetti G., Signorelli M.S., Fusar Poli L., Gesi C., Aguglia E. (2019). Sex Differences in the Relationship between PTSD Spectrum Symptoms and Autistic Traits in a Sample of University Students. Clin. Pract. Epidemiol. Ment. Health.

[14] Sucksmith E., Roth I., Hoekstra R.A. (2011). Autistic traits below the clinical threshold: Re-examining the broader autism phenotype in the 21st century. Neuropsychol. Rev..

[15] Takara K., Kondo T. (2014). Comorbid atypical autistic traits as a potential risk factor for suicide attempts among adult depressed patients: A case—Control study. Ann. Gen. Psychiatry.

[16] Dell’Osso L., Carpita B., Gesi C., Cremone I.M., Corsi M., Massimetti E., Muti D., Calderani E., Castellini G., Luciano M. (2018). Subthreshold autism spectrum disorder in patients with eating disorders. Compr. Psychiatry.

[17] Dell’Osso L., Carpita B., Bertelloni C.A., Diadema E., Barberi F.M., Gesi C., Carmassi C. (2019). Subthreshold autism spectrum in bipolar disorder: Prevalence and clinical correlates. Psychiatry Res..

[18] Dell’Osso L., Carpita B., Muti D., Morelli V., Salarpi G., Salerni A., Scotto J., Massimetti G., Gesi C., Ballerio M. (2019). Mood symptoms and suicidality across the autism spectrum. Compr. Psychiatry.

[19] Dell’Osso L., Conversano C., Corsi M., Bertelloni C.A., Cremone I.M., Carpita B., Carbone M.G., Gesi C., Carmassi C. (2018). Polysubstance and behavioral addictions in a patient with bipolar disorder: Role of lifetime subthreshold autism spectrum. Case Rep. Psychiatry.

[20] Kato K., Mikami K., Akama F., Yamada K., Maehara M., Kimoto K., Kimoto K., Sato R., Takahashi Y., Fukushima R. (2013). Clinical features of suicide attempts in adults with autism spectrum disorders. Gen. Hosp. Psychiatry.

[21] Hollocks M.J., Lerh J.W., Magiati I., Meiser-Stedman R., Brugha T.S. (2019). Anxiety and depression in adults with autism spectrum disorder: A systematic review and meta-analysis. Psychol. Med..

[22] Kõlves K., Fitzgerald C., Nordentoft M., Wood S.J., Erlangsen A. (2021). Assessment of suicidal behaviors among individuals with autism spectrum disorder in Denmark. JAMA Netw. Open.

[23] Carpita B., Carmassi C., Calderoni S., Muti D., Muscarella A., Massimetti G., Cremone I.M., Gesi C., Conti E., Muratori F. (2020). The broad autism phenotype in real-life: Clinical and functional correlates of autism spectrum symptoms and rumination among parents of patients with autism spectrum disorder. CNS Spectr..

[24] Dell’Osso L., Cremone I.M., Carpita B., Fagiolini A., Massimetti G., Bossini L., Vita A., Barlati S., Carmassi C., Gesi C. (2018). Correlates of autistic traits among patients with borderline personality disorder. Compr. Psychiatry.

[25] Pelton M.K., Cassidy S.A. (2017). Are autistic traits associated with suicidality? A test of the interpersonal-psychological theory of suicide in a non-clinical young adult sample. Autism Res..

[26] Baron-Cohen S., Ring H.A., Bullmore E.T., Wheelwright S., Ashwin C., Williams S.C. (2000). The amygdala theory of autism. Neurosci. Biobehav. Rev..

[27] Salazar F., Baird G., Chandler S., Tseng E., O’sullivan T., Howlin P., Pickles A., Simonoff E. (2015). Co-occurring Psychiatric Disorders in Preschool and Elementary School-Aged Children with Autism Spectrum Disorder. J. Autism Dev. Disord..

[28] Vickerstaff S., Heriot S., Wong M., Lopes A., Dossetor D. (2007). Intellectual ability, self-perceived social competence, and depressive symptomatology in children with high-functioning autistic spectrum disorders. J. Autism Dev. Disord..

[29] Hallett V., Lecavalier L., Sukhodolsky D.G., Cipriano N., Aman M.G., McCracken J.T., McDougle C.J., Tierney E., King B.H., Hollander E. (2013). Exploring the manifestations of anxiety in children with autism spectrum disorders. J. Autism Dev. Disord..

[30] White S.W., Bray B.C., Ollendick T.H. (2012). Examining shared and unique aspects of Social Anxiety Disorder and Autism Spectrum Disorder using factor analysis. J. Autism Dev. Disord..

[31] Pugliese C.E., White B.A., White S.W., Ollendick T.H. (2013). Social anxiety predicts aggression in children with ASD: Clinical comparisons with socially anxious and oppositional youth. J. Autism Dev. Disord..

[32] Postorino V., Kerns C.M., Vivanti G., Bradshaw J., Siracusano M., Mazzone L. (2017). Anxiety Disorders and Obsessive-Compulsive Disorder in Individuals with Autism Spectrum Disorder. Curr. Psychiatry Rep..

[33] Afif I.Y., Farkhan M., Kurdi O., Maula M.I., Ammarullah M.I., Setiyana B., Jamari J., Winarni T.I. (2022). Effect of Short-Term Deep-Pressure Portable Seat on Behavioral and Biological Stress in Children with Autism Spectrum Disorders: A Pilot Study. Bioengineering.

[34] Lai M.C., Baron-Cohen S. (2015). Identifying the lost generation of adults with autism spectrum conditions. Lancet Psychiatry.

[35] Green R.M., Travers A.M., Howe Y., McDougle C.J. (2019). Women and Autism Spectrum Disorder: Diagnosis and Implications for Treatment of Adolescents and Adults. Curr. Psychiatry Rep..

[36] Attwood T. (2007). The Complete Guide to Asperger’s Syndrome.

[37] Gould J., Ashton-Smith J. (2011). Missed diagnosis or misdiagnosis? Girls and women on the autism spectrum. GAP.

[38] Kopp S., Gillberg C. (2011). The autism spectrum screening questionnaire (ASSQ)-revised extended version (ASSQ-REV): An instrument for better capturing the autism phenotype in girls? A preliminary study involving 191 clinical cases and community controls. Res. Dev. Disabil..

[39] Lai M.C., Lombardo M.V., Pasco G., Ruigrok A.N.V., Wheelwright S.J., Sadek S.A., Chakrabarti B., Baron-Cohen S., MRC AIMS Consortium (2011). A behavioral comparison of male and female adults with high functioning autism spectrum conditions. PLoS ONE.

[40] Cook J., Hull L., Crane L., Mandy W. (2021). Camouflaging in autism: A systematic review. Clin. Psychol. Rev..

[41] Lai M.C., Lombardo M.V., Ruigrok A.N., Chakrabarti B., Auyeung B., Szatmari P., Happé F., Baron-Cohen S., MRC AIMS Consortium (2017). Quantifying and exploring camouflaging in men and women with autism. Autism.

[42] Hull L., Petrides K.V., Allison C., Smith P., Baron-Cohen S., Lai M.C., Mandy W. (2017). “Putting on My Best Normal”: Social Camouflaging in Adults with Autism Spectrum Conditions. J. Autism Dev. Disord..

[43] Dell’Osso L., Lorenzi P., Carpita B. (2021). Camouflaging: Psychopathological meanings and clinical relevance in autism spectrum conditions. CNS Spectr..

[44] Duvekot J., van der Ende J., Verhulst F.C., Slappendel G., van Daalen E., Maras A., Greaves-Lord K. (2017). Factors influencing the probability of a diagnosis of autism spectrum disorder in girls versus boys. Autism.

[45] Dworzynski K., Ronald A., Bolton P., Happé F. (2012). How different are girls and boys above and below the diagnostic threshold for autism spectrum disorders?. J. Am. Acad. Child Adolesc. Psychiatry.

[46] Head A.M., McGillivray J.A., Stokes M.A. (2014). Gender differences in emotionality and sociability in children with autism spectrum disorders. Mol. Autism.

[47] Kirkovski M., Enticott P.G., Fitzgerald P.B. (2013). A review of the role of female gender in autism spectrum disorders. J. Autism Dev. Disord..

[48] Whitlock A., Fulton K., Lai M.C., Pellicano E., Mandy W. (2020). Recognition of Girls on the Autism Spectrum by Primary School Educators: An Experimental Study. Autism Res..

[49] Van Wijngaarden-Cremers P.J.M., van Eeten E., Groen W.B., Van Deurzen P.A., Oosterling I.J., Van der Gaag R.J. (2014). Gender and age differences in the core triad of impairments in autism spectrum disorders: A systematic review and meta-analysis. J. Autism Dev. Disord..

[50] Kreiser N.L., White S.W. (2014). ASD in females: Are we overstating the gender difference in diagnosis?. Clin. Child Fam. Psychol. Rev..

[51] Carpita B., Cremone I.M., Amatori G., Cappelli A., Salerni A., Massimetti G., Borgioli D., Carmassi C., Massai R., Dell’Osso L. (2022). Investigating the relationship between orthorexia nervosa and autistic traits in a university population. CNS Spectr..

[52] Lehnhardt F.G., Falter C.M., Gawronski A., Pfeiffer K., Tepest R., Franklin J., Vogeley K. (2016). Sex-Related Cognitive Profile in Autism Spectrum Disorders Diagnosed Late in Life: Implications for the Female Autistic Phenotype. J. Autism Dev. Disord..

[53] Cola M.L., Plate S., Yankowitz L., Petrulla V., Bateman L., Zampella C.J., de Marchena A., Pandey J., Schultz R.T., Parish-Morris J. (2020). Sex differences in the first impressions made by girls and boys with autism. Mol. Autism.

[54] Newschaffer C.J., Croen L.A., Daniels J., Giarelli E., Grether J.K., Levy S.E., Mandell D.S., Miller L.A., Pinto-Martin J., Reaven J. (2007). The epidemiology of autism spectrum disorders. Annu. Rev. Public Health.

[55] Nicholas J.S., Charles J.M., Carpenter L.A., King L.B., Jenner W., Spratt E.G. (2008). Prevalence and characteristics of children with autism-spectrum disorders. Ann. Epidemiol..

[56] Begeer S., Mandell D., Wijnker-Holmes B., Venderbosch S., Rem D., Stekelenburg F., Koot H.M. (2013). Sex differences in the timing of identification among children and adults with autism spectrum disorders. J. Autism Dev. Disord..

[57] Giarelli E., Wiggins L.D., Rice C.E., Levy S.E., Kirby R.S., Pinto-Martin J., Mandell D. (2010). Sex differences in the evaluation and diagnosis of autism spectrum disorders among children. Disabil. Health J..

[58] Rutherford M., McKenzie K., Johnson T., Catchpole C., O’Hare A., McClure I., Forsyth K., McCartney D., Murray A. (2016). Gender ratio in a clinical population sample, age of diagnosis and duration of assessment in children and adults with autism spectrum disorder. Autism.

[59] Shattuck P.T., Durkin M., Maenner M., Newschaffer C., Mandell D.S., Wiggins L., Lee L.C., Rice C., Giarelli E., Kirby R. (2009). Timing of identification among children with an autism spectrum disorder: Findings from a population-based surveillance study. J. Am. Acad. Child Adolesc. Psychiatry.

[60] Schuck R.K., Flores R.E., Fung L.K. (2019). Brief Report: Sex/Gender Differences in Symptomology and Camouflaging in Adults with Autism Spectrum Disorder. J. Autism Dev. Disord..

[61] Boyd K., Woodbury-Smith M., Szatmari P. (2011). Managing anxiety and depressive symptoms in adults with autism-spectrum disorders. J. Psychiatry Neurosci..

[62] Simone R. (2010). Aspergirls: Empowering Females with Asperger Syndrome.

[63] Williams D. (1992). Nobody Nowhere: The Remarkable Autobiography of an Autistic Girl.

[64] Bargiela S., Steward R., Mandy W. (2016). The Experiences of Late-diagnosed Women with Autism Spectrum Conditions: An Investigation of the Female Autism Phenotype. J. Autism Dev. Disord..

[65] Dell’Osso L., Carpita B., Lorenzi P. (2019). Autistic traits and illness trajectories. Clin. Pract. Epidemiol. Ment. Health.

[66] Lai M.C., Lombardo M.V., Auyeung B., Chakrabarti B., Baron-Cohen S. (2015). Sex/gender differences and autism: Setting the scene for future research. J. Am. Acad. Child Adolesc. Psychiatry.

[67] Livingston L.A., Happé F. (2017). Conceptualising compensation in neurodevelopmental disorders: Reflections from autism spectrum disorder. Neurosci. Biobehav. Rev..

[68] Cage E., Di Monaco J., Newell V. (2018). Experiences of Autism Acceptance and Mental Health in Autistic Adults. J. Autism Dev. Disord..

[69] Tierney S., Burns J., Kilbey E. (2016). Looking behind the mask: Social coping strategies of girls on the autistic spectrum. Res. Autism Spectr. Disord..

[70] Dean M., Harwood R., Kasari C. (2017). The art of camouflage: Gender differences in the social behaviors of girls and boys with autism spectrum disorder. Autism.

[71] Cassidy S., Bradley L., Shaw R., Baron-Cohen S. (2018). Risk markers for suicidality in autistic adults. Mol. Autism.

[72] Hull L., Mandy W., Lai M.C., Baron-Cohen S., Allison C., Smith P., Petrides K.V. (2019). Development and Validation of the Camouflaging Autistic Traits Questionnaire (CAT-Q). J. Autism Dev. Disord..

[73] Dell’Osso L., Cremone I.M., Muti D., Massimetti G., Lorenzi P., Carmassi C., Carpita B. (2022). Validation of the Italian version of the Camouflaging Autistic Traits Questionnaire (CAT-Q) in a University population. Compr. Psychiatry.

[74] Hull L., Lai M.C., Baron-Cohen S., Allison C., Smith P., Petrides K.V., Mandy W. (2020). Gender differences in self-reported camouflaging in autistic and non-autistic adults. Autism.

[75] Cassidy S.A., Gould K., Townsend E., Pelton M., Robertson A.E., Rodgers J. (2020). Is Camouflaging Autistic Traits Associated with Suicidal Thoughts and Behaviours? Expanding the Interpersonal Psychological Theory of Suicide in an Undergraduate Student Sample. J. Autism Dev. Disord..

[76] Cage E., Troxell-Whitman Z. (2019). Understanding the Reasons, Contexts and Costs of Camouflaging for Autistic Adults. J. Autism Dev. Disord..

[77] Beck J.S., Lundwall R.A., Gabrielsen T., Cox J.C., South M. (2020). Looking good but feeling bad: “Camouflaging” behaviors and mental health in women with autistic traits. Autism.

[78] Robinson E., Hull L., Petrides K.V. (2020). Big Five model and trait emotional intelligence in camouflaging behaviours in autism. Personal. Individ. Differ..

[79] Perry E., Mandy W., Hull L., Cage E. (2022). Understanding Camouflaging as a Response to Autism-Related Stigma: A Social Identity Theory Approach. J. Autism Dev. Disord..

[80] Hull L., Levy L., Lai M.C., Petrides K.V., Baron-Cohen S., Allison C., Smith P., Mandy W. (2021). Is social camouflaging associated with anxiety and depression in autistic adults?. Mol. Autism.

[81] Hull L., Petrides K.V., Mandy W. (2021). Cognitive Predictors of Self-Reported Camouflaging in Autistic Adolescents. Autism Res..

[82] Dell’Osso L., Cremone I.M., Chiarantini I., Arone A., Massimetti G., Carmassi C., Carpita B. (2022). Autistic traits and camouflaging behaviors: A cross-sectional investigation in a University student population. CNS Spectr..

[83] McQuaid G.A., Lee N.R., Wallace G.L. (2022). Camouflaging in autism spectrum disorder: Examining the roles of sex, gender identity, and diagnostic timing. Autism.

[84] Milner V., Mandy W., Happé F., Colvert E. (2022). Sex differences in predictors and outcomes of camouflaging: Comparing diagnosed autistic, high autistic trait and low autistic trait young adults. Autism.

[85] Westwood H., Eisler I., Mandy W., Leppanen J., Treasure J., Tchanturia K. (2016). Using the autism-spectrum quotient to measure autistic traits in anorexia nervosa: A systematic review and meta-analysis. J. Autism Dev. Disord..

[86] Dell’Osso L., Cremone I.M., Carpita B., Dell’Oste V., Muti D., Massimetti G., Barlati S., Vita A., Fagiolini A., Carmassi C. (2019). Rumination, posttraumatic stress disorder, and mood symptoms in borderline personality disorder. Neuropsychiatr. Dis. Treat..

[87] Carpita B., Muti D., Cremone I.M., Fagiolini A., Dell’Osso L. (2022). Eating disorders and autism spectrum: Links and risks. CNS Spectr..

[88] Dell’Osso L., Carpita B. (2022). What misdiagnoses do women with autism spectrum disorder receive in the DSM-5?. CNS Spectr..

[89] Dell’Osso L., Cremone I.M., Amatori G., Cappelli A., Cuomo A., Barlati S., Massimetti G., Vita A., Fagiolini A., Carmassi C. (2021). Investigating the Relationship between Autistic Traits, Ruminative Thinking, and Suicidality in a Clinical Sample of Subjects with Bipolar Disorder and Borderline Personality Disorder. Brain Sci..

[90] Dell’Osso L., Muti D., Carpita B., Cremone I.M., Bui E., Gesi C., Carmassi C. (2018). The Adult Autism Subthreshold Spectrum (AdAS) model: A neurodevelopmental approach to mental disorders. J. Psychopathol..

[91] Hannon B., Mandy W., Hull L. (2023). A comparison of methods for measuring camouflaging in autism. Autism Res..

